# Anti-proliferative impact of resveratrol on gingival fibroblasts from juvenile hyaline fibromatosis

**DOI:** 10.1007/s00784-024-05771-7

**Published:** 2024-07-26

**Authors:** Işıl Saygun, Melis Özgül Slezovic, Cansel Köse Özkan, Vahdi Umut Bengi, Pınar Elçi, Muhittin Serdar, Alpdogan Kantarci

**Affiliations:** 1 Department of Periodontology, Gulhane Faculty of Dental Medicine, University of Health Sciences, Ankara, Turkey; 2 Gulhane Faculty of Pharmacy, University of Health Sciences, Ankara, Turkey; 3 Gulhane Health Sciences Institute, Stem Cell Lab, University of Health Sciences, Ankara, Turkey; 4 Department of Medical Biochemistry, Acıbadem Mehmet Ali Aydınlar University, Ankara, Turkey; 5 Forsyth Institute, Cambridge, MA USA

**Keywords:** Resveratrol, Gingival enlargement, Juvenile hyaline fibromatosis, CTGF, TGF- β, Collagen

## Abstract

**Aim:**

Resveratrol is a natural polyphenolic compound with biological activities such as anti-inflammation and antioxidation. Its anti-fibrotic effect has been experimentally demonstrated in the pancreas and liver. This study aims to determine the anti-proliferative effect of resveratrol on fibroblasts obtained from hyperplastic gingival tissues from a patient diagnosed with Juvenile Hyaline Fibromatosis (JHF).

**Materials and methods:**

Primary gingival fibroblast cell lines were obtained from gingival growth tissues by the gingivectomy of a patient with JHF. Gingival fibroblasts were treated with or without 3 different doses of resveratrol (50, 100, 200 µM). Cytotoxicity and cell proliferation were evaluated after 24, 48, and 72 h. Collagen, TGF, and CTGF were analyzed by ELISA in the 48-hour supernatants.

**Results:**

All three doses of resveratrol suppressed the proliferation of JHF gingival fibroblasts at 24 and 48 h without showing any cytotoxic effect compared to the control group (*p* < 0.0001). At 72 h, 100 and 200 µM resveratrol showed significantly less proliferation (*p* < 0.0001), less collagen, CTGF, and TGF- β (*p* < 0.001) than the control group.

**Conclusion:**

Resveratrol had a profound anti-proliferative effect on gingival fibroblasts obtained from gingival enlargements with JHF, suggesting that it can be used as a therapeutic to prevent excessive cell growth by suppressing collagen, CTGF, and TGF- β synthesis in the pathogenesis of hyperplasia.

## Introduction

Gingival fibromatoses are fibrous proliferations of the gingiva that can be caused by idiopathic, hereditary, genetic, or other diseases or syndromes [1]. Juvenile hyaline fibromatosis (JHF) is a genetic condition that is characterised by the accumulation of amorphous hyaline material, nodular tumours of the skin, gingival enlargement, osteolytic lesions and joint contractures [2, 3]. Although the origin of this amorphous hyaline material is not known, it appears to contain glycoproteins, glycosaminoglycans, and collagens [4, 5].

It is thought that impaired collagen synthesis may be a significant contributing factor in the development of this disease [3]. The research has focused on fibrosis-related factors, including cellular proliferation, collagen synthesis, extracellular matrix degeneration, cytokines, and growth factors [6]. Various agents can regulate fibroblast proliferation, including the cytokine transforming growth factor-beta1 (TGF-β1) stimulation [7]. TGF- β, a pluripotent cytokine with a well-known role in fibrogenesis processes involved in regulating ECM remodeling by inducing collagen turnover regulation, may affect the overall balance of collagen deposition [7, 8].

The connective tissue growth factor (CTGF) is a matricellular factor associated with fibrosis essential in producing and maintaining fibrotic lesions [9]. CTGF is highly expressed in fibrotic lesions such as skin and kidney fibrosis and atherosclerosis [10–12]. As TGF-β1 potently induces CTGF in fibroblastic cells from a variety of tissues, the cooperative effect of both types of fibrogenic cytokines has been suggested to contribute to the regulation of fibrosis [10–13].

The main structural component of the gingival connective tissue is collagen I (Col I), which is involved in wound healing processes and connective tissue remodeling; however, its excessive production can lead to extracellular matrix accumulation and, eventually, tissue fibrosis [14, 15].

Excessive gingival enlargement at a very early age in JHF patients affects the eruption of both primary and permanent teeth [16]. The young age of the patients makes the surgical treatment of overgrowth more complicated, and at the same time, the high role of recurrence requires the development of alternative treatment modalities or adjunctive therapy for surgery.

The search for methods using non-invasive natural products as an alternative to the surgical treatment of gingival enlargements has continued. In recent years, interest in their potential use as an adjunct to treating inflammatory diseases and conditions, including periodontitis, has increased since the pharmacological properties of polyphenols were identified [17]. Resveratrol is a polyphenolic compound found in grapes, wine, peanuts, and blueberries and may positively affect various chronic inflammatory conditions, tumor or cancer development, and apoptotic diseases [18, 19]. Furthermore, studies suggest that resveratrol has an anti-fibrotic role in fibrogenic and epithelial-mesenchymal processes in organs such as the pancreas and liver [20, 21].

The aim of this study was to evaluate whether resveratrol has an anti-proliferative effect by suppressing collagen, TGF- β, and CTGF levels, which have been shown to play a role in the etiology of gingival enlargement in gingival fibroblasts isolated from gingival growths with JHF in cell culture.

## Materials and methods

### Study design

The present study was approved by the ethics committee of Gülhane Scientific Research and Health Science University. This study was carried out in the laboratory of the Stem Cell Research Unit of the University of Health Sciences Gulhane Institute Research and Development Center.

A 22-year-old female patient with JHF who had systemic involvement and gingival enlargements and was followed for 20 years, gingivectomy operation was performed on the overgrowth areas after obtaining consent. Gingival tissues to be removed and discarded after gingivectomy were frozen and stored for use as JHF cell lines with the consent of the patient. The gingival tissues were placed in a 14 mm falcon containing 5 mm DMEM (Dulbecco Modified Eagle Medium) containing 1%, 100% U/ml penicillin and 100 µg/ml streptomycin and transported to the laboratory environment. The gingival tissues placed into the petri dish were cut into little pieces in the laboratory using a 22-size scalpel. The cut pieces were inoculated into a 25-gauge flask with a sterile syringe tip. 10% medium (10% fetal calf serum − 1% penicillin − 1% L glutamine) was added and placed in an oven containing 5% CO2 at 37 ºC to be immobilized for 72 h. When it became apparent that cells were growing around the tissue, the medium with the same properties was refreshed. This procedure was repeated every 72 h until all cells covered the flask (80% area). Then, trypsinization was performed, and the cells were transferred to a large 75-well flask. This way, sufficient cells were reached, and cell lines were frozen and stored.

**Fig. 1:**
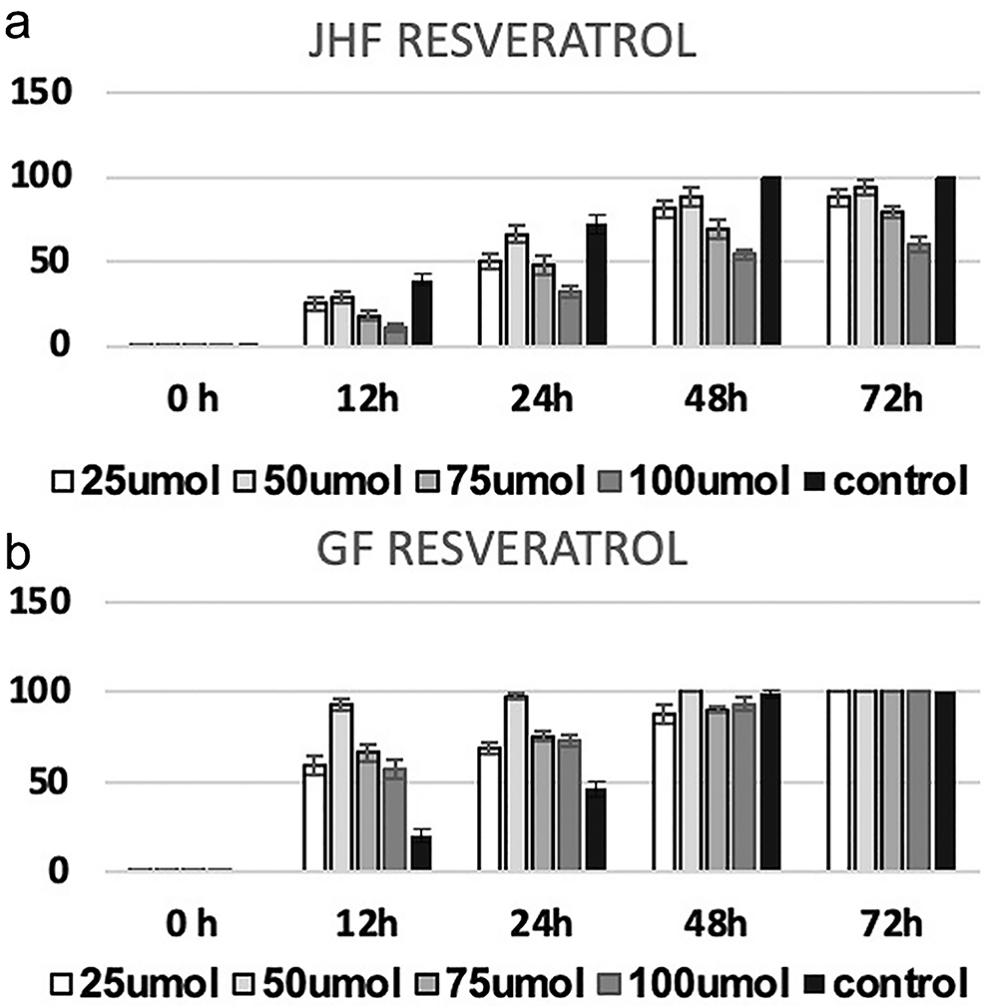
**(a)**: In JHF cells; 24, 48 and 72 h mean and standard deviation values of cell indexes of the control group without resveratrol and the groups applied with different doses of resveratrol (50, 75, 100 mmol resveratrol). While all doses showed significantly less proliferation than the control, the anti-proliferative effect of the 100 Mm resveratrol dose was especially evident (*p* < 0.0001). (Control: JHF cells without resveratrol) **(b)**: In healthy gingival fibroblast cells; 24, 48 and 72 h mean and standard deviation values of (GF) cell indexes in the control group without resveratrol and the groups applied with different doses of resveratrol (50,75,100 mmol resveratrol). There was no significant difference between the groups at any time period (*p* > 0.05) [22]

**Fig. 2:**
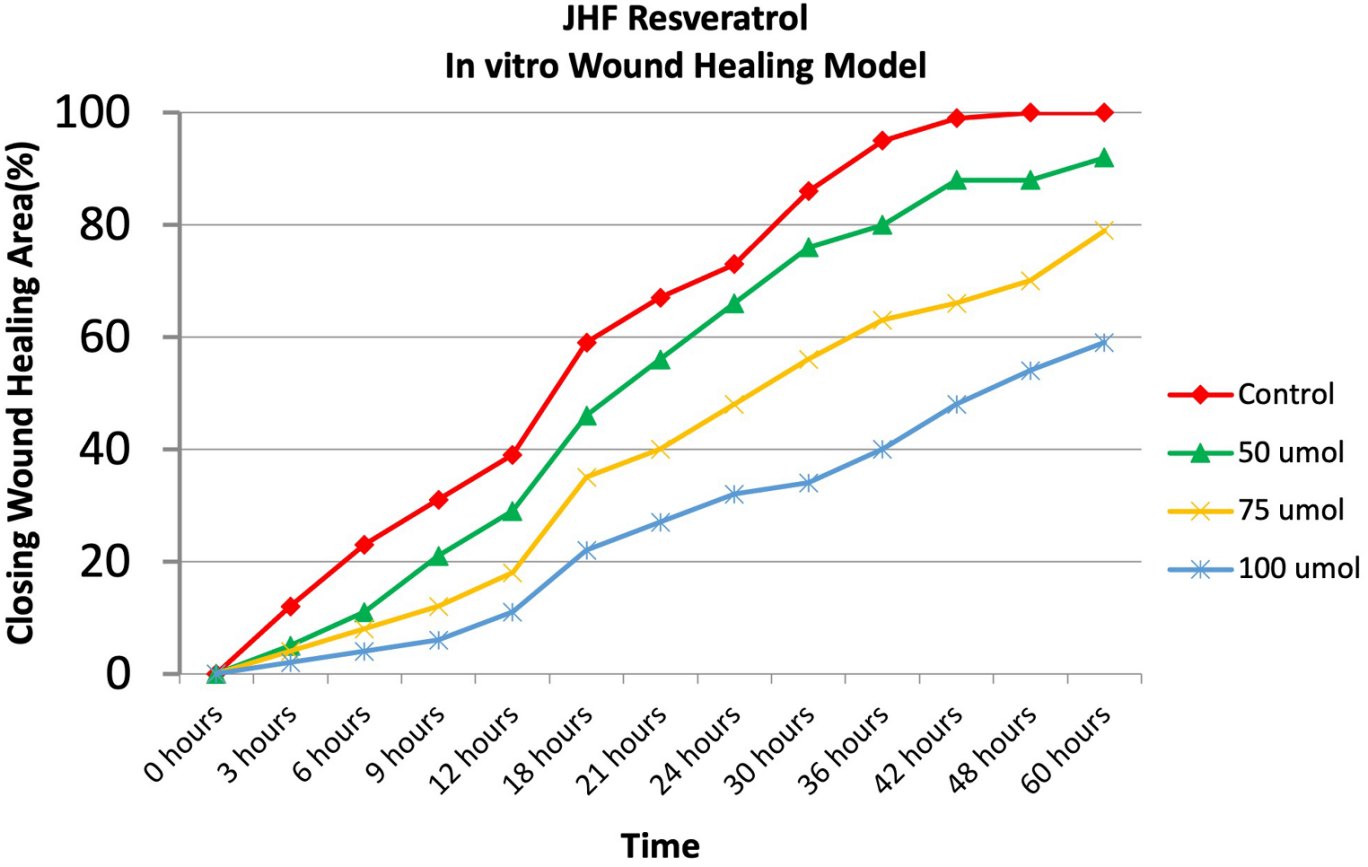
As a result of comparing the control group without resveratrol and the groups with different doses of resveratr ol (50,75,100 mmol resveratrol) in a wound model, it was shown that all resveratrol doses showed anti-proliferative effects, but 100µM resveratrol was the most effective dose (*p* < 0.0001) (Control: JHF cells without resveratrol) [22]

In previously our study [22], gingival fibroblasts (GF) were obtained from the gingival tissues removed from systemically healthy patients who received dental implants during the healing cap installation phase. These cells constituted the healthy control group (GF). Human gingival fibroblasts harvested from the JHF patient constituted the JHF group. Informed consent was obtained from patients. Primary cultures of JHF and GF cells were incubated with different concentrations of resveratrol. Cell proliferation was assessed by real time cell analysis system xcelligence impedance method and wound healing model (Figs. 1 and 2) [22]. Fibroblast cells were monitored every 30 min for a period of upto 72 h by the xCELLigence system. In this study [22], the effect of three different concentrations of resveratrol (50 µM, 75 µM and 100 µM), selected according to real-time cell impedance evaluation results, on wound healing was evaluated compared to a control group without resveratrol. Every 12 h, the appearance of the wound shape was captured with a digital camera. Photography was completed when all of the cells covered the wound area. Photographs were analyzed with a Java-based Image J computer program. In our previous study [22], we found that 100 µM resveratrol was the optimal dosage for inhibiting proliferation in JHF cells until the 60th hour (Fig. 2) [22]. Based on this dosage, lesser and higher doses were added to the research. Frozen and stored cells were thawed for study, and 24 well plates were seeded with 5x${10}^{4}$ cells in each well. Resveratrol was included in the standard media at doses prepared in the test groups.

### Specifications and preparation of resveratrol

The active substance, resveratrol, used in our study was analyzed using Fourier transform infrared (FTIR) spectroscopy to determine its specifications. Approximately 100 mg of Resveratrol Sigma (Lot 038K5202, USA) was placed between the crystal and the compression apparatus of a Perkin Elmer FTIR instrument, and the spectrum was obtained in the range of 650–4000 ${\text{c}\text{m}}^{-1}$. For cell culture experiments, resveratrol stock solution was prepared under sterile conditions in a Class II laboratory. 45.6 mg of Resveratrol (Sigma R5010, USA) was dissolved in 1 ml of sterile alcohol. The solution was then passed through a 0.45-micron filter and diluted to 10 ml with cell culture medium, resulting in a 20 mmol stock solution. For the experiments, three different doses of resveratrol (50 µM, 100 µM, 200 µM) were prepared from this stock solution as needed.

### Cell viability and proliferation

The effects of different doses of resveratrol 50 μm, 100 μm, 200 μm on the viability and cell proliferation of gingival fibroblasts with JHF and control fibroblasts with JHF without resveratrol were evaluated by MTT assay.

MTT is a water-soluble tetrazolium salt and is a yellow-colored solution. As a result of the cleavage of the tetrazolium ring by dehydrogenase enzymes, MTT turns into an orange-soluble formazan. Dehydrogenase enzymes are present in the mitochondria of living cells; dead cells cannot perform this change. Formazan concentration is directly proportional to the number of viable cells. The resulting color reaction is read spectrophotometrically at a wavelength of 570 nm, and absorbance values are determined [23]. For this purpose, JHF fibroblasts were seeded into 96-well plates at ${10}^{4}$ cells/well density and cultured at 37 ºC under 5% CO2 pressure for 24 h. After 24 h, 100 µl of doses of resveratrol (50,100, 200 µl) were added to each well (only 100 µl media for control wells). Then, each well received 20 µl of MTT solution (Sigma, Germany), and the cells were incubated at 37 °C for 4 h. Then the culture medium and MTT solution were aspirated and replaced by DMSO solution to dissolve the formazan. At the end of the procedure, the absorbance at the plate was gently shaken and read in an ELISA Reader at 570 nm wavelength. These procedures were performed at all resveratrol doses for the 24th, 48th, and 72nd hours.

### ELISA assay

The levels of COL-1, TGF- β, and CTGF (Fine Test Human TGF- β, CTGF, COL-1 Wuhan Biotech Co., Ltd., Wuhan, Hubei, China) in the supernatants of each culture were determined using commercially available ELISA kits. Samples were frozen at − 80 $℃$ until analysis. One day before the study, the samples were taken to + 4 and thawed slowly. COL-1, TGF- β, and CTGF levels in the supernatants of the experimental groups administered 2 different doses of resveratrol with the best anti-proliferative effect, and the control groups not administered resveratrol were examined by ELISA at 48 h according to the manufacturer’s protocol (Fine Test Human TGF- β, CTGF, COL-1 Wuhan Biotech Co., Ltd., Wuhan, Hubei, China). Each sample was assessed in triplicate.

### Statistical analysis

Statistical analysis was performed with SPSS Statistics Version 22 (IBM Corp., Armonk, NY, USA). Shapiro-Wilk test was used to evaluate whether the data had a normal distribution. Kruskal Wallis test was used to test the significance of the difference between the means of the doses in MTT evaluations. Analysis of Variance (ANOVA) was used to determine whether the growth factor release values of the study groups differed from each other. A significant difference was found between the groups with ANOVA. Tukey-Kramer test, one of the post-hoc tests, was used to determine which groups differed. When evaluating the results of the tests, differences below *p* < 0.05 were noted as significant.

## Results

We previously showed resveratrol treatment could has an anti-proliferative effects on JHF cells while this effect is not observed in healthy gingival fibroblasts (Figs. 1 and 2). Within doses using in this study, 100 µM RVT had revealed best anti-proliferative effect JHF cells (Figs. 1a and 2) [22]. Therefore, In this study, we investigated the potential and mechanisms of resveratrol in reducing proliferation in JHFs.

### MTT results

Our previous study found that 100 µM resveratrol was the optimal dosage for inhibiting proliferation in JHF cells until the 60th hour (Fig. 2) [22]. In the new research presented based on this dosage, lower and higher doses were added.

**Fig. 3:**
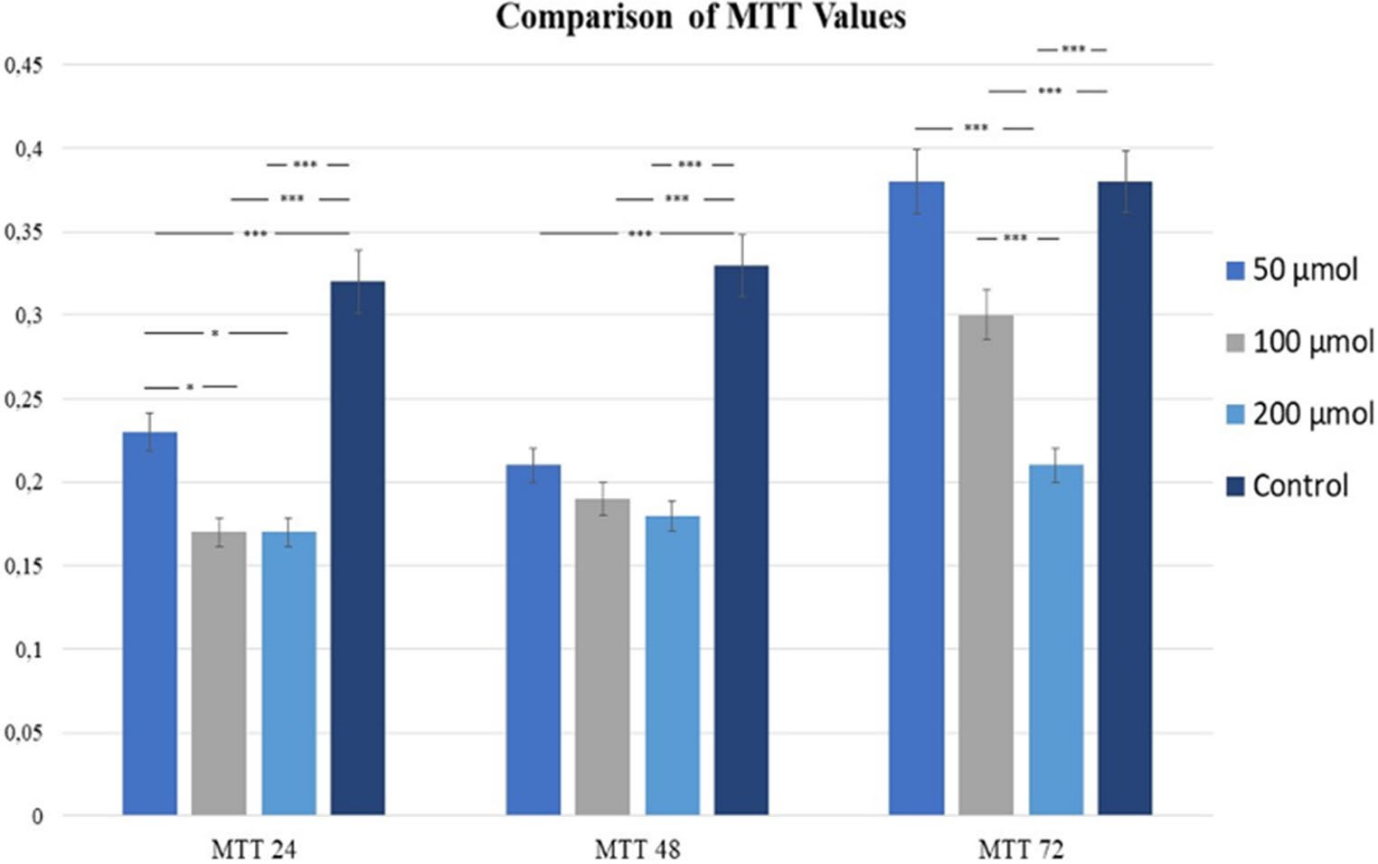
The effect of resveratrol on JHF cells in the control group without resveratrol and in the groups administered 100 μm and 200 μm resveratrol was evaluated using the MTT proliferation assay at 24, 48 and 72 h. *p < 0.5 **p < 0.01,***p < 0.001 was considered significantly different, Control: JHF cells without resveratrol

In this study, 3 different doses of resveratrol 50 μm, 100 μm, 200 μm in gingival fibroblasts with JHF suppressed proliferation at the 24th hour, without showing any cytotoxic effect at all 3 doses when compared to cells with JHF without resveratrol in the control group (Fig. 3). Although there was no significant difference between 100 μm and 200 μm resveratrol, both groups showed significantly less proliferation than 50 μm resveratrol (*p* < 0.05). At the 48th hour, 50 μm, 100 μm and 200 μm resveratrol cells showed significantly lower proliferation than control group cells (*p* < 0.0001), and at 72 h, 100 μm and 200 μm resveratrol cells showed significantly less proliferation compared to control group cells (*p* < 0.0001), but no significant difference was found between 50 μm resveratrol and control. 200 μm resveratrol cells showed lower proliferation values than the cells in 100 μm resveratrol (*p* < 0.0001) (Fig. 3).

### ELISA results

**Fig. 4:**
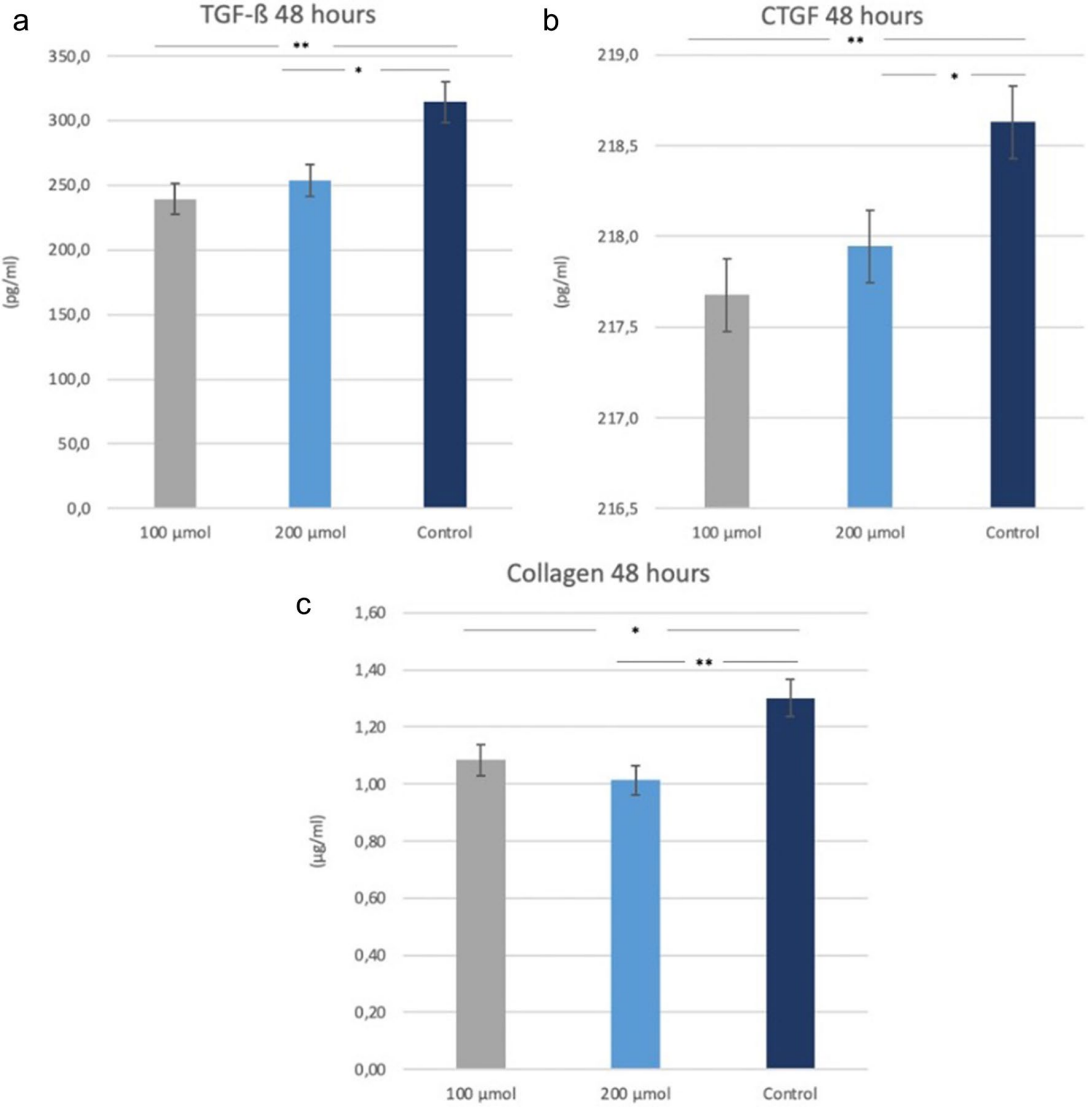
In JHF cells; TGF-β (a), CTGF (b) and Collagen (c) levels were measured in cell culture supernatants via ELISA assay in the control group without resveratrol and in the groups administered 100 μm and 200 μm resveratrol. *p < 0.05, ** *p* < 0.01 was considered significantly different (Control: JHF cells without resveratrol)

Considering the expression values of growth factors, both 100 μm and 200 μm resveratrol cells synthesized significantly less TGF-β and CTGF than the control group cells at the 48th hour (*p* < 0.0001) **(**Fig. 4a, b) There was no significant difference between the two doses of resveratrol regarding CTGF and TGF-β expression of gingival fibroblasts with JHF. When looking at the collagen expression in the study groups, at 48 h, both 100 μm and 200 μm resveratrol-treated cells with JHF synthesized significantly less collagen than the control group JHF fibroblasts without resveratrol (respectively, *p* = 0.049, *p* = 0.0083) (Fig. 4c).

## Discussion

In this study, it was aimed to evaluate whether resveratrol, a natural polyphenolic compound with various biological activities such as anti-inflammation and antioxidation, has an anti-proliferative effect on fibroblasts obtained from overgrown gingival tissues of Juvenil Hyaline Fibromatosis patient in cell culture. It was observed that 3 different doses of resveratrol (50,100,200 μm) used in JHF cells suppressed proliferation at the 24th and 48th hours without showing any cytotoxic effect compared to the control group JHF fibroblast cells. At 72nd hours, 100 μm and 200 μm resveratrol cells showed significantly less proliferation compared to the control, while no significant difference was observed between 50 μm and the control groups.

Previous studies on gingival fibromatosis reported an autocrine role of TGF- β as a stimulator of fibroblast proliferation [24–27]. Gawron et al. reported that CTGF, TGF- β and type 1 collagen levels increased in hereditary gingival fibromatosis tissues when compared with healthy tissues in their in vitro study [15]. Similarly, Uzel et al. observed increased CTGF levels in drug-induced gingival enlargement and tissues with hereditary gingival fibromatosis patients [27]. Since CTGF and TGF- β have been shown to play a role in the pathogenesis of gingival fibromatosis in previous studies [15, 28–30], the expression values of these growth factors and cell proliferation values were evaluated in our study. We observed that resveratrol suppressed CTGF and TGF- β levels expressed in JHF cells at 100 and 200 μm doses.

No study in the literature shows the effect of resveratrol in gingival fibromatosis within the scope of dentistry. Hereby, we were able to compare the results of our study with studies in medicine examining the effect of resveratrol on fibrotic diseases. Ahmad et al. suggested that resveratrol protects tissues from oxidative damage and provides benefits for fibrotic conditions in the liver [31]. The anti-fibrotic effect of Resveratrol was investigated by Tsang et al. using the rat pancreatic stellate cellular model, and it was shown to have potent anti-fibrotic activities. More importantly, administration of transresveratrol (10–50 mM) significantly reduced TGF- β -stimulated AKT phosphorylation [19]. Bai et al. reported that resveratrol could be a potential therapeutic to prevent progressive renal tubulointerstitial fibrosis by suppressing TGF- β expression [20]. Li et al. reported that resveratrol inhibited liver fibrosis induced by activation of TGFβ/Smad3 signaling pathways [32]. Another study reported that the anti-fibrotic effects of resveratrol partly by reducing the expression of the profibrogenic cytokine TGF-β and inhibiting TGF-β–Smad-3 signaling [33]. In our study, it was observed that doses of 100 and 200 μm resveratrol suppressed TGF- β expression in a statistically significant way in fibroblasts produced from gingival overgrowth tissue with JHF, and these results suggested that resveratrol may have a therapeutic effect in the prevention of gingival fibromatosis in patients with JHF.

Oxidative stress occurs when there is an imbalance between the production of reactive oxygen species (ROS) and the capacity of the cells’ antioxidant clearance mechanisms [34]. Previous studies have shown that oxidative stress may play a critical role in the initiation and progression of fibrotic diseases [35, 36] and a relationship between oxidative stress and gingival enlargement. Becerik et al. observed changes in oxidative stress markers for cyclosporine-induced gingival overgrowth [37]. Consistent with similar studies [34, 38, 39], Malo et al. observed that collagen synthesis was significantly increased by exposure of herediter gingival fibroblasts to H2O2, an oxidant, and that this effect could be reversed by the addition of CoQ10, an antioxidant. It has shown for the first time that collagen synthesis is affected by an oxidant and can be restored by an antioxidant in fibroblasts [40]. Our study observed that resveratrol, an antioxidant, can suppress collagen synthesis, similar to CoQ10 in Malo et al.‘s study. Deng et al. showed that increased production of ROS in gingival fibroblasts induced TGF- β activation and increased the synthesis of connective tissue growth factor [41]. Similarly, Chen et al. demonstrated in vitro that an increase in ROS can lead to the development of gingival overgrowth [42]. However, the use of Curcumin, an antioxidant, can block TGF-β1-induced CTGF expression on gingival fibroblasts. In this study, it was determined that resveratrol, which has antioxidant properties, suppressed autocrine CTGF synthesis in fibroblasts obtained from JHF tissues.

Resveratrol has also been shown to reduce inflammation by inhibiting prostaglandin production and cyclooxygenase-2 activity [43]. The anti-inflammatory effect of resveratrol may contribute to its anti-fibrotic effect [44]. It suggests that inflammation due to dental plaque plays an important role in the etiology of gingival enlargements induced by drugs and various genetic factors. Thus, the anti-inflammatory property of resveratrol may provide additional contributions to the treatment of gingival enlargements. It is planned to examine the anti-inflammatory properties of resveratrol in prospective clinical studies.

One of the limitations of this study is the use of only non-resveratrol-treated gingival fibroblasts with JHF as the control group. These cells are the cells that were previously frozen and thawed during the experiment. To evaluate the effect of resveratrol, we used these cells as a control group instead of the new primary healthy control group. In the in vitro study we conducted when these JHF cells were first produced, we saw that the proliferation was higher than that of the healthy cells. However, since we used frozen cells in this study, we could not evaluate with healthy controls.

It is thought that resveratrol may be helpful in the anti-fibrotic treatment of cases of gingival fibromatosis, Further experimental and clinical studies are needed to determine the dosage and effect of resveratrol better. In future studies, we aim to investigate local or systemic applications of resveratrol with controlled release systems or suitable carriers to develop a non-invasive adjuvant treatment for gingival hyperplasia.

## Conclusions

In this study, resveratrol showed a dose-dependent anti-proliferative effect in gingival fibroblasts obtained from gingival enlargements with JHF. It was thought that resveratrol could achieve this effect by suppressing collagen, CTGF, and TGF- β synthesis, which is thought to play a role in the pathogenesis of hyperplasia, by preventing excessive cell growth.

Our study provides important data for developing new, non-invasive strategies aimed at restoring gingival tissue homeostasis for the treatment of enlargement in patients with JHF in the future.

## Data Availability

No datasets were generated or analysed during the current study.
